# Hyperglycaemia is a causal risk factor for upper limb pathologies

**DOI:** 10.1093/ije/dyad187

**Published:** 2024-01-10

**Authors:** Harry D Green, Ella Burden, Ji Chen, Jonathan Evans, Kashyap Patel, Andrew R Wood, Robin N Beaumont, Jessica Tyrrell, Timothy M Frayling, Andrew T Hattersley, Richard A Oram, Jack Bowden, Inês Barroso, Christopher Smith, Michael N Weedon

**Affiliations:** Department of Clinical and Biomedical Sciences, University of Exeter, Exeter, UK; Shoulder Unit, Princess Elizabeth Orthopaedic Centre, Royal Devon and Exeter Hospital, Exeter, UK; Department of Clinical and Biomedical Sciences, University of Exeter, Exeter, UK; Shoulder Unit, Princess Elizabeth Orthopaedic Centre, Royal Devon and Exeter Hospital, Exeter, UK; Department of Clinical and Biomedical Sciences, University of Exeter, Exeter, UK; Department of Clinical and Biomedical Sciences, University of Exeter, Exeter, UK; Department of Clinical and Biomedical Sciences, University of Exeter, Exeter, UK; Department of Clinical and Biomedical Sciences, University of Exeter, Exeter, UK; Department of Clinical and Biomedical Sciences, University of Exeter, Exeter, UK; Department of Clinical and Biomedical Sciences, University of Exeter, Exeter, UK; Department of Clinical and Biomedical Sciences, University of Exeter, Exeter, UK; Department of Clinical and Biomedical Sciences, University of Exeter, Exeter, UK; Department of Clinical and Biomedical Sciences, University of Exeter, Exeter, UK; Shoulder Unit, Princess Elizabeth Orthopaedic Centre, Royal Devon and Exeter Hospital, Exeter, UK; Department of Clinical and Biomedical Sciences, University of Exeter, Exeter, UK

**Keywords:** Diabetes, hyperglycaemia, musculoskeletal, frozen shoulder, Dupuytren’s, trigger finger, carpal tunnel syndrome, causality, mendelian randomization

## Abstract

**Background:**

Diabetes (regardless of type) and obesity are associated with a range of musculoskeletal disorders. The causal mechanisms driving these associations are unknown for many upper limb pathologies. We used genetic techniques to test the causal link between glycemia, obesity and musculoskeletal conditions.

**Methods:**

In the UK Biobank’s unrelated European cohort (*N* = 379 708) we performed mendelian randomisation (MR) analyses to test for a causal effect of long-term high glycaemia and adiposity on four musculoskeletal pathologies: frozen shoulder, Dupuytren’s disease, carpal tunnel syndrome and trigger finger. We also performed single-gene MR using rare variants in the *GCK* gene.

**Results:**

Using MR, we found evidence that long-term high glycaemia has a causal role in the aetiology of upper limb conditions. A 10-mmol/mol increase in genetically predicted haemoglobin A1C (HbA1c) was associated with frozen shoulder: odds ratio (OR) = 1.50 [95% confidence interval (CI), 1.20–1.88], Dupuytren’s disease: OR = 1.17 (95% CI, 1.01–1.35), trigger finger: OR = 1.30 (95% CI, 1.09–1.55) and carpal tunnel syndrome: OR = 1.20 (95% CI, 1.09–1.33). Carriers of *GCK* mutations have increased odds of frozen shoulder: OR = 7.16 (95% CI, 2.93–17.51) and carpal tunnel syndrome: OR = 2.86 (95% CI, 1.50–5.44) but not Dupuytren’s disease or trigger finger. We found evidence that an increase in genetically predicted body mass index (BMI) of 5 kg/m^2^ was associated with carpal tunnel syndrome: OR = 1.13 (95% CI, 1.10–1.16) and associated negatively with Dupuytren’s disease: OR = 0.94 (95% CI, 0.90–0.98), but no evidence of association with frozen shoulder or trigger finger. Trigger finger (OR 1.96 (95% CI, 1.42–2.69) *P *=* *3.6e-05) and carpal tunnel syndrome [OR 1.63 (95% CI, 1.36–1.95) *P *=* *8.5e-08] are associated with genetically predicted unfavourable adiposity increase of one standard deviation of body fat.

**Conclusions:**

Our study consistently demonstrates a causal role of long-term high glycaemia in the aetiology of upper limb musculoskeletal conditions. Clinicians treating diabetes patients should be aware of these complications in clinic, specifically those managing the care of *GCK* mutation carriers. Upper limb musculoskeletal conditions should be considered diabetes complications.

Key Messages

- Glycaemia is a causal risk factor for frozen shoulder, Dupuytren’s disease, trigger finger and carpal tunnel syndrome.
- Higher body mass index is a causal risk factor for carpal tunnel syndrome but a causal protective factor for Dupuytren’s disease.
- Clinicians managing the treatment of individuals with diabetes should be aware of the important role of raised glycaemia in the development of upper limb pathologies

## Introduction

Diabetes, regardless of type, is known to cause a number of micro- and macrovascular complications for which there are good awareness and monitoring.^1^ However, there is increasing recognition of associated musculoskeletal complications.^2^ Causation of these musculoskeletal complications has not previously been demonstrated, and there is no current routine monitoring for these conditions in people with diabetes.

Observational epidemiological studies have found associations of type 1 and type 2 diabetes, and obesity, with a range of fibroproliferative musculoskeletal conditions of the upper limb that clinically manifest as limited joint mobility. Larkin *et al.* proposed that the following conditions should be considered ‘diabetic cheiroarthropathies’: adhesive capsulitis (frozen shoulder), Dupuytren’s disease, flexor tenosynovitis (trigger finger), carpal tunnel syndrome and positive prayer sign^3^ (although the term cheiroarthropathy is more commonly used to refer exclusively to diabetic stiff hand in the musculoskeletal literature).^4^ Larkin *et al.* further described an association between these five pathologies and age, sex, diabetes duration and haemoglobin A1c (HbA1c), but did not consider body mass index (BMI). BMI associates observationally with frozen shoulder, trigger finger and carpal tunnel syndrome in the UK Biobank cohort,^5^^,^^6^ and Dupuytren’s disease is well known to inversely associate with BMI.^7^ However, the causal mechanisms behind these observational associations is unclear.

In our recent work we demonstrated, using mendelian randomization (MR), that type 1 diabetes is a causal risk factor for frozen shoulder, and hypothesized that this association is explained by long-term raised glycaemia as a result of type 1 diabetes.^5^ MR is a technique in genetic epidemiology that is roughly analogous to a randomized controlled trial, where people are randomized at conception to certain genotypes. It allows evaluating the effect of an exposure (e.g. BMI) on an outcome (e.g. trigger finger) by using genetic variants as ‘instruments’ for the exposure. Because genotypes are assigned at conception, they do not suffer from ‘reverse causality’ (i.e. BMI cannot change a person’s genotype) and so it is a useful tool to assert causality based on cross-sectional data.

Other studies using MR to ascertain the causality of risk factors for upper limb musculoskeletal pathologies are sparse. Higher BMI has previously been shown to be a causal risk factor for Dupuytren’s disease,^8^ but also shown to have a protective causal effect on the risk of Dupuytren’s disease.^9^^,^^10^ The causal impact of obesity on frozen shoulder and trigger finger, and the causal impact of high glycaemia on musculoskeletal conditions, have not yet been demonstrated.

Here, we first used MR to test whether glycaemia (measured by blood glucose levels and by HbA1c) and obesity (measured by BMI, waist-hip-ratio and body fat percentage) are causal risk factors for fibroproliferative upper limb musculoskeletal pathologies. We also used rare genetic variants associated with blood glucose levels as an alternative, single-gene MR instrument.

## Methods

### Selection and definition of outcomes

We studied the following four pathologies: frozen shoulder, Dupuytren’s disease, trigger finger and carpal tunnel syndrome, defined using codes in Supplementary Table S1 (available as Supplementary data at *IJE* online). We derived two definitions for each outcome: a broad definition to maximize sample size (using the International Classification of Diseases, 10th Revision (ICD-10), Operating Procedure Codes Supplement 4 (OPCS4), general practice (GP) and self-report data) and a specific definition using only Hospital Episode Statistics (HES) with greater case certainty and likely with higher case severity (ICD-10 and OPCS4 only). We do not study positive prayer sign, as the ICD-10 code R29.8 (Other and unspecified symptoms and signs involving the nervous and musculoskeletal systems) is not specific to positive prayer sign. Data on total numbers of cases can be found in Supplementary Table S2 (available as Supplementary data at *IJE* online).

### Exposure selection and definition

To test for a causal role of blood sugar level on musculoskeletal conditions, we used HbA1c and blood glucose levels recorded by the UK Biobank. These were defined using data fields 30750 and 30740, respectively, in the UK Biobank’s baseline data. Individuals missing these fields were excluded from the respective analyses.

We tested for a causal role of obesity on musculoskeletal conditions using BMI defined using data field 21001. As carpal tunnel syndrome is associated with adipose tissue in the carpal canal, obesity-related associations may be driven by overall adiposity.^12^ In addition to BMI, we also tested waist-hip ratio (WHR) calculated using data fields 48 (waist circumference) and 49 (hip circumference) and body fat percentage (BFP) defined using data field 23099.

Our genetic instruments for HbA1c were obtained from the Meta-Analysis of Glucose and Insulin-related Traits Consortium (MAGIC). These variants were identified in a multi-ancestry meta-analysis, and a fuzzy clustering approach was used to determine whether they influence HbA1c through a glycaemic or a non-glycaemic pathway.^13^ We used the 51 single nucleotide polymorphisms (SNPs), identified to affect HbA1c through a glycaemic pathway, to isolate a glycaemic effect for our genetic instrument for HbA1c, and as a sensitivity, also tested the 124 SNPs identified to affect HbA1c through a non-glycaemic pathway. We use the 130 SNPs for fasting blood glucose, based on the same meta-analysis, as an instrument for blood glucose levels.

The genetic variants extracted for BMI and our genetic risk score (GRS) for BMI used a 2015 genome-wide association study (GWAS) of 339 224 individuals that reported 97 genome-wide significant loci.^14^ We excluded sex-specific variants and those with potential pleiotropy or secondary signals within a locu, and used 72 variants.^15^ We used two different sets of variants and GRS for WHR, both derived from a meta-analysis of 694 649 individuals of European ancestry.^16^ For BFP, we used 36 variants for favourable and 39 unfavourable adiposity.^17^

All studies used to derive genetic instruments other than favourable/unfavourable adiposity did not use the UK Biobank. This minimizes overlap between samples used for genotype-exposure and genotype-outcome associations. See Supplementary Table S3 (available as Supplementary data at *IJE* online) for a full list of variants used.

### Statistical methods

All analyses in the UK Biobank were performed on the unrelated European population, defined using a principal components analysis (PCA). A KING Kinship matrix was used to exclude those third-degree relatives or closer.^18^ An optimal list of unrelated individuals was generated by preferentially removing individuals with the maximum number of relatives, to allow the maximum number of individuals to be included (*N* = 379 713).

All subsequent statistical analysis was performed using R 4.0.3 and compiled into a Word document with RMarkdown.

#### Observational evidence

We tested for an association between musculoskeletal conditions and exposure traits using a logistic regression model adjusted for age and sex. To test for non-linearity in the relationship between exposure traits and musculoskeletal outcomes, we also performed logistic regression using a spline with five knots. Effect sizes are reported as odds ratio per standard deviation increase in exposure trait. This analysis was performed in the UK Biobank cohort.

#### One-sample mendelian randomization evidence

We performed one-sample MR to test for causality by combining the genetic instruments into a genetic risk score (GRS), using the published effect sizes for each SNP as weights. First, a linear regression was performed using a normalized GRS and the exposure. This regression was adjusted for the first five genetic principal components. The second stage was also linear, regressing the normalized predicted value against the outcome trait, adjusted for the residuals, age and sex. We adjusted all effect sizes to report odds ratios on the same scales as the observational analysis. This analysis was performed in the UK Biobank cohort.

MR methods are subject to the following assumptions:

- relevance: the genetic instrument associates with the exposure;
- exclusion restriction: the genetic instrument only affects the outcome via the relationship with the exposure;
- independence: the genetic instrument is independent of confounders on the exposure-outcome relationship.

#### Two-sample mendelian randomization evidence

We further used the IVW (inverse variance weighted) test and MR Egger^19^ two-sample mendelian randomization methods to test for causality. Two-sample mendelian randomization approaches regress the genotype-exposure effect sizes for a set of genetic instruments against the genotype-outcome effect sizes.

For the genotype-outcome associations, we first performed a GWAS in the UK Biobank using BOLT-LMM.^20^ Then, we used the publicly available statistics from FinnGen, a Finnish population of 218 792 individuals, to run the same analysis. In FinnGen, we used the variables M13_ADHCAPSULITIS, M13_DUPUTRYEN, M13_TRIGGERFINGER and G6_CARPTU for frozen shoulder, Dupuytren’s disease, trigger finger and carpal tunnel syndrome, respectively. Finally, we meta-analysed the results of UK Biobank and FinnGen using betas and standard errors, and used this meta-analysed GWAS for the genotype-outcome associations. Analyses of favourable and unfavourable adiposity were performed only in FinnGen because the genetic variants were derived in the UK Biobank.

#### Glucokinase variants

Finally, we tested the association of each condition with individuals who had a pathogenic *GCK* variant using Fisher’s exact test. Pathogenic *GCK* variants result in stable lifelong raised fasting glucose levels. Pathogenic *GCK* variants were defined as described in Mirshahi *et al*.^21^ Briefly, these were either protein-truncating variants or missense variants that had been previously reported to be pathogenic and occurred <2 in gnomAD (https://gnomad.broadinstitute.org/).

### Ethics approval and data availbility

Ethics approval for the UK Biobank study was obtained from the North West Centre for Research Ethics Committee (11/NW/0382).^11^ Written informed consent was obtained from all participants. All individual-level data used in this paper were obtained from the UK Biobank resource, and can be obtained from the UK Biobank at [https://www.ukbiobank.ac.uk/enable-your-research/apply-for-access]. Information on recruitment, locations and data collection methods can be found in Bycroft *et al*.^11^ Access to summary statistics from the FinnGen resource can be obtained at [https://www.finngen.fi/en/access_results/].

## Results

### Observational associations for upper limb musculoskeletal conditions with adiposity and glycaemic traits

Observational associations of BMI and glycaemic traits for each condition are shown in Table 1. Dupuytren’s disease is much more common in men, but frozen shoulder, trigger finger and carpal tunnel syndrome are more common in women. All four conditions associate observationally with HbA1c and glucose, adjusted for age and sex. Frozen shoulder, trigger finger, and carpal tunnel syndrome associate with adiposity-related traits (BMI, WHR, body fat percentage). Dupuytren’s disease associated negatively with these traits. All conditions associated with smoking. Only Dupuytren’s disease showed a strong association with alcohol consumption. These results were consistent when GP and self-report cases were included (Supplementary Tables S4 and S5, available as Supplementary data at *IJE* online).

**Table 1.** Observational associations with musculoskeletal conditions

	Frozen shoulder		Dupuytren’s disease		Trigger finger		Carpal tunnel syndrome	
Trait	OR (95% CI)	*P*	OR (95% CI)	*P*	OR (95% CI)	*P*	OR (95% CI)	*P*
Sex (*n* = 379 713)	0.74 (0.67–0.81)	4.9e-11	3.68 (3.42–3.96)	2.1e-262	0.75 (0.70–0.81)	1.9e-14	0.51 (0.49–0.53)	1.8e-206
Age (*n* = 379 713)	1.00 (1.00–1.01)	0.79	1.09 (1.08–1.09)	9.4e-251	1.04 (1.04–1.05)	5e-60	1.03 (1.02–1.03)	2.1e-86
HbA1c (*n* = 361 887)	1.24 (1.20–1.27)	9.7e-56	1.04 (1.01–1.07)	0.0027	1.20 (1.17–1.22)	1.1e-52	1.22 (1.21–1.24)	1.3e-172
Glucose (*n* = 331 248)	1.20 (1.18–1.23)	4.4e-55	1.04 (1.01–1.07)	0.0047	1.14 (1.11–1.17)	4.4e-26	1.13 (1.11–1.15)	9.1e-58
BMI (*n* = 378 214)	1.15 (1.1–1.19)	3.4e-11	0.76 (0.73–0.79)	1.4e-42	1.23 (1.2–1.27)	7.4e-40	1.46 (1.44–1.48)	0
WHR (*n* = 378 974)	1.23 (1.17–1.3)	6.5e-14	0.91 (0.87–0.95)	3.6e-05	1.20 (1.15–1.26)	5.2e-16	1.47 (1.43–1.51)	1.9e-195
BFP (*n* = 378 974)	1.34 (1.26–1.42)	3.5e-21	0.88 (0.84–0.92)	3.6e-08	1.12 (1.06–1.17)	9.8e-06	1.32 (1.29–1.36)	2.8e-92
Smoking (*n* = 317 754)	1.14 (1.09–1.19)	4.2e-09	1.08 (1.05–1.11)	6.9e-07	1.15 (1.11–1.19)	1e-15	1.19 (1.17–1.21)	1.6e-67
Alcohol (*n* = 150 972)	0.92 (0.85–1.00)	0.057	1.22 (1.17–1.27)	9.2e-22	0.95 (0.89–1.01)	0.12	0.95 (0.91–0.99)	0.011

All conditions associated with HbA1c, glucose level, BMI, WHR, BF and smoking status (although Dupuytren’s disease notably associated negatively for BMI, WHR and BF). Only Dupuytren’s disease associated with alcohol intake. All associations are logistic regression-adjusted for age and sex (except age and sex, which were univariable).

BFP, body fat percentage; BMI, body mass index; HbA1c, haemoglobin A1c; OR, odds ratio; WHR, waist-hip ratio.

### Hyperglycaemia causes upper limb musculoskeletal conditions

**Figure 1.**
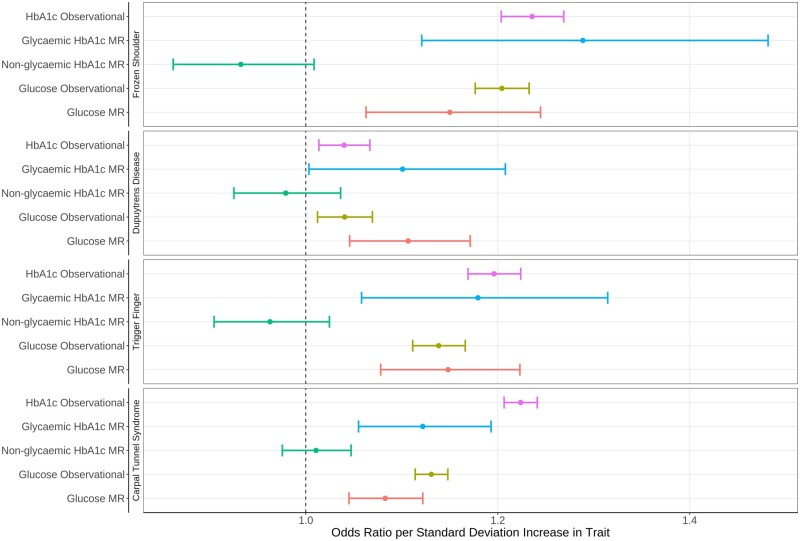
MR shows strong evidence for a causal role of long-term high glycaemia on all four conditions. Glycaemic HbA1c MR and non-glycaemic HbA1c MR refer to MR performed using the SNPs identified by the MAGIC consortium to raise HbA1c through glycaemic and non-glycaemic pathways, respectively. Glucose uses fasting glucose SNPs identified by MAGIC. Conditions defined by ICD-10 and OPCS4 codes. MR, mendelian randomization; HbA1c, haemoglobin A1c; SNP, single nucleotide polymorphism; ICD-10, International Classification of Diseases, 10th Revision; OPCS4, Operating Procedure Codes Supplement 4

Figure 1 and Supplementary Table S6 (available as Supplementary data at *IJE* online) show the observational and magnetic resonance (MR) imaging results per standard deviation increase in HbA1c and blood glucose levels. The strong observational associations for HbA1c were supported by MR using SNPs acting on glycaemic pathways. There was no evidence of any association with SNPs acting through the non-glycaemic pathways. Blood glucose was consistently associated with all four conditions in both the observational and MR analyses. In the Supplementary figures we show the non-linear relationship between HbA1c (Supplementary Figure S1, available as Supplementary data at *IJE* online), blood glucose levels (Supplementary Figure S2, available as Supplementary data at *IJE* online) and BMI (Supplementary Figure S3, available as Supplementary data at *IJE* online).

The causal estimates for HbA1c equate to an odds ratio per 10-mmol/mol increase in HbA1c of 1.50 (95% CI, 1.20–1.88); *P *=* *0.00037, for frozen shoulder, 1.17 (95% CI, 1.01–1.35); *P = *0.042, for Dupuytren’s disease, 1.30 (95% CI, 1.09–1.55); *P = *0.0029, for trigger finger and 1.20 (95% CI, 1.09–1.33); *P = *0.00025, for carpal tunnel syndrome.

All GRSs were strong instrumental variables. The F statistics for the genetic risk scores were 2189 for HbA1c through glycaemic pathways, 8590 for HbA1c through other pathways and 4048 for blood glucose.

These results were consistent when GP records and self-report data were used (Supplementary Table S7, available as Supplementary data at *IJE* online). In the two-sample meta-analysed results with FinnGen using the IVW test, frozen shoulder 2.44 (95% CI, 1.84–3.24); *P = *1.3e-09, Dupuytren’s disease 2.87 (95% CI, 2.25–3.67); *P = *2.8e-16, trigger finger 3.19 (95% CI, 2.39–4.26); *P = *3e-14, and carpal tunnel syndrome 2.14 (95% CI, 1.82–2.52); *P = *1.1e-18, associated with HbA1c. Full two-sample MR (2SMR) results from each data source can be found in Supplementary Table S8 (available as Supplementary data at *IJE* online), and the full meta-analysed results including fasting glucose and Egger test can be found in Supplementary Table S9 (available as Supplementary data at *IJE* online).

In all 139 of the unrelated cohort were carriers of pathogenic *GCK* mutations. These mutations associated with frozen shoulder and carpal tunnel syndrome (Table 2). These pathogenic mutations strongly associated with an increase in HbA1c (HbA1c mmol/mol) and glucose (glucose mmol/L), but not obesity-related traits, smoking or alcohol (Supplementary Table S10, available as Supplementary data at *IJE* online).

**Table 2.** Associations between *GCK* mutations and musculoskeletal conditions

	HES		All records	
Condition	OR (95% CI)	*P*	OR (95% CI)	*P*
Frozen shoulder	7.16 (2.93–17.51)	1.6e-05	4.25 (2.35–7.69)	1.7e-06
Dupuytren’s disease	2.89 (1.07–7.8)	0.037	2.26 (1.00–5.12)	0.051
Trigger finger	1.76 (0.44–7.12)	0.43	0.92 (0.23–3.72)	0.91
Carpal tunnel syndrome	2.86 (1.50–5.44)	0.0014	2.12 (1.18–3.84)	0.013

*GCK* mutations strongly associated with frozen shoulder and carpal tunnel syndrome when conditions were defined by HES codes. Evidence for an association between Dupuytren’s disease and GCK mutations was limited. All associations were attenuated when self-report and general practice records were included in the condition definition.

HES, Hospital Episode Statistics.

### Mixed results for causal impact of obesity and adiposity-related traits on musculoskeletal conditions

**Figure 2.**
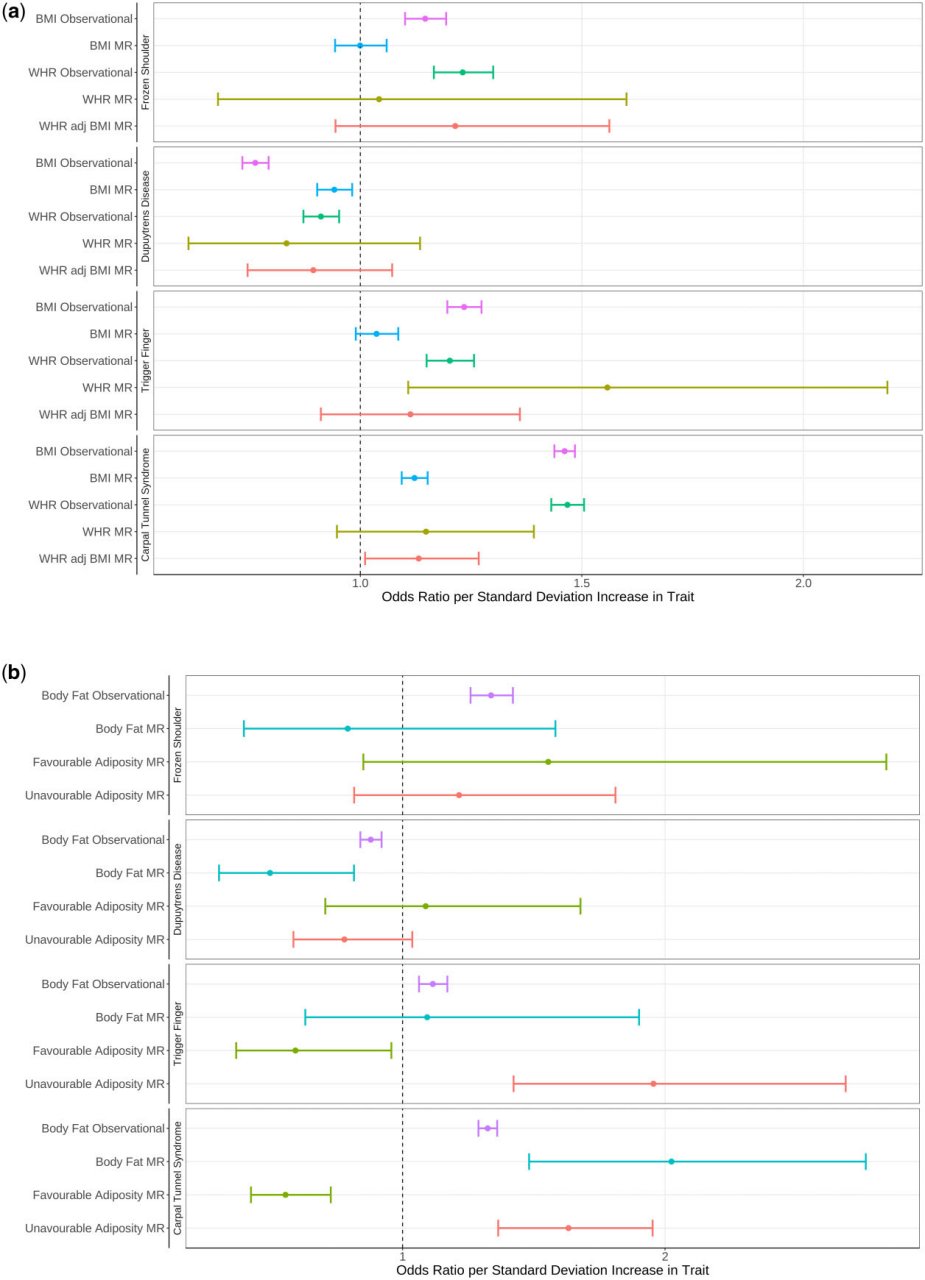
a) Mendelian randomization shows evidence that raised body mass index has a causal increase in risk of carpal tunnel syndrome and a causal reduction in risk of Dupuytren’s disease. b) Mendelian randomization of increased body fat shows the same association as body mass index. Favourable/unfavourable adiposity represent increased body fat percent accompanied by a favourable/unfavourable metabolic profile. Unfavourable adiposity has a causal role in trigger finger and carpal tunnel syndrome. BMI, body mass index; WHR, waist-hip ratio

Figure 2a and Supplementary Table S4 (available as Supplementary data at *IJE* online) show the observational and MR results per standard deviation increase in BMI and WHR. The strong observational associations of frozen shoulder with BMI and WHR were not observed in MR analysis. The negative association of BMI with Dupuytren’s disease was consistent in MR, but WHR was not. Trigger finger associated with BMI and WHR observationally, but only the WHR association was consistent with the observational effect. The observational association of BMI with carpal tunnel syndrome was attenuated in the MR result of 1.46 (95% CI, 1.44–1.48) vs 1.12 (95% CI, 1.09–1.15) increase per SD increase in BMI.

Similar results showing body fat measurements are shown in Figure 2b and Supplementary Table S4 (available as Supplementary data at *IJE* online). Observational associations of body fat with and frozen shoulder and trigger finger were not observed in MR analysis. Body fat percentage had a negative effect on risk of Dupuytren’s disease and a positive effect on risk of carpal tunnel syndrome, which was consistent in both observational and MR analyses. Trigger finger and carpal tunnel syndrome further associated negatively with favourable adiposity and positively with unfavourable adiposity.

All GRSs were strong instrumental variables. The F statistics were 5834 for BMI, 3296 for WHR, 9786 for WHR adjusted for BMI, 1219 for body fat, 1605 for favourable adiposity and 3764 for unfavourable adiposity.

The results were repeated in the two-sample MR meta-analysis: Dupuytren’s disease associated negatively with BMI at −0.26 (95% CI, −0.36 to −0.15); *P = *3e-06, whereas carpal tunnel syndrome at 0.51 (95% CI, 0.44–0.57); *P = *7.9e-47., and trigger finger at 0.25 (95% CI, 0.12–0.38); *P = *0.00021, associated positively, and frozen shoulder at 0.02 (95% CI, −0.10 to 0.14); *P = *0.75, did not associate. Full 2SMR results from each data source can be found in Supplementary Table S8 (available as Supplementary data at *IJE* online), and the full meta-analysed results, including fasting waist-hip ratio, body fat and favourable adiposity, alongside Egger and penalized weighted median (PWM) test results can be found in Supplementary Table S9 (available as Supplementary data at *IJE* online).

## Discussion

### Principal findings

This study investigated the aetiological basis of long-term hyperglycaemia and obesity on the development of musculoskeletal conditions in a large European population. We tested the causality of hyperglycaemia using HbA1c and glucose levels, and obesity using BMI, WHR and body fat. We found strong evidence of a causal role of hyperglycaemia on all four conditions: frozen shoulder, Dupuytren’s disease, trigger finger and carpal tunnel syndrome, which was consistent observationally, and in one- and two-sample MR. We also showed that individuals with pathogenic mutations in the *GCK* gene are at increased risk of musculoskeletal conditions. Evidence for obesity was mixed: whereas all conditions associated observationally with BMI and waist-hip-ratio, in the MR analysis we only found strong evidence of a causal role of obesity on carpal tunnel syndrome and a protective effect on Dupuytren’s disease. The glycaemic associations from MR were consistent with the observational associations, but the obesity associations were attenuated compared with the observational result. It was previously well known that diabetes is associated with musculoskeletal conditions,^3^ and our results provide further insight into this link, establishing that there is a causal link through glycaemia.

### Interpretation

Advanced glycation end products are raised in the palm muscles of Dupuytren’s disease patients^22^ and in the shoulders of frozen shoulder patients,^23^ and associate with tendon thickness, decreased shoulder mobility and upper extremity function.^14^ Our results suggest that this is causal, and the causal mechanism behind the upper limb pathologies studied is likely raised glycation end products as a result of long-term high glycaemia. Other than a much stronger association with frozen shoulder and *GCK* mutations compared with the other conditions, we see very few differences in the results for each condition, suggesting that all share a similar glycaemic aetiology with respect to glucose.

It has previously been hypothesized that the protective effect of high BMI on Dupuytren’s disease are due to negative genetic correlations between these phenotypes.^24^ Our results for BMI also show a protective effect, but due to the less precise estimate, our causal estimate for WHR adjusted for BMI was compatible with both a strong negative effect and a null effect. Previous studies have explained the BMI and carpal tunnel syndrome association by higher BMI leading to increased fat deposit in the carpal canal and increased pressure in the carpal tunnel.^12^ BMI-defined obesity and frozen shoulder were associated in some studies.^25^ Our results suggest that BMI associates observationally with frozen shoulder due to confounding with HbA1c. The MR estimate for BMI was not compatible with the observational estimate. Similar to frozen shoulder, trigger finger associated with BMI in previous studies, but we find no evidence of a causal association.^26^

### Strengths and weaknesses

The main strength of our research is the large sample size and depth of information available in the UK Biobank resource. Starting with 500 000 people, we identified 2015 cases of frozen shoulder, 4129 of Dupuytren’s disease, 1989 of trigger finger and 10 654 of carpal tunnel syndrome. The availability of linked genetic data allows us to conduct studies of causality using one- and two-sample Mendelian randomization-based approaches, and the availability of the FinnGen resource allowed us to validate the two-sample findings in a second cohort. We study multiple exposures for glycaemic and obesity-related traits, and performed MR using both common variants and monogenic variants to minimize the possibility of confounding in any particular result, leading to invalid conclusions.

We acknowledge several limitations of using a cross-sectional sample like the UK Biobank. First, the recruitment age was between 37 and 73 years with a bias towards healthy individuals. Further, we stratified the data to only include people of European ancestry, so results may not be generalizable to other ethnic groups. Self-report and GP data are potentially unreliable data sources; however, our results were consistent when using just the HES records. Our causal estimates come from genetics, which cause a change throughout the individual’s life. As such, we are only able to infer a causal association from long-term high glycaemia (caused by genetics) and cannot ascertain the causality of short-term interventions (such as treatment or lifestyle changes).

Whereas the most recent and largest study of the genetic architecture of glycaemia (from the MAGIC consortium) reports genetic variants for fasting glucose and 2-h glucose tolerance, the closest phenotype recorded at baseline in the UK Biobank was random glucose measurements. As such in the one-sample MR results, the first stage of our 2SLS regression regressed a GRS for fasting blood glucose against random glucose measurements, which will weaken the strength of the genetic instrument and could cause weak instrument bias. However, this bias is not present in the two-sample MR, for which the SNP exposure estimates come from MAGIC rather than UK Biobank.

### Conclusion

The majority of work on diabetes complications is centred on macro- and microvascular conditions. Here, we have demonstrated evidence that four musculoskeletal conditions that are more common in people with diabetes are caused by elevated blood glucose levels. Further research in diabetes complications should consider musculoskeletal conditions alongside more established complications such as heart and kidney disease. Clinicians treating people with diabetes, particularly those managing the care of *GCK* mutation carriers, should be aware of these complications.

## Ethics approval

Ethics approval for the UK Biobank study was obtained from the North West Centre for Research Ethics Committee (11/NW/0382).^11^ Written informed consent was obtained from all participants.

## Supplementary Material

dyad187_Supplementary_Data

## Data Availability

All individual-level data used in this paper were obtained from the UK Biobank resource, and can be obtained from the UK [iobank at [https://www.ukbiobank.ac.uk/enable-your-research/apply-for-access]. Information on recruitment, locations and data collection methods can be found in Bycroft *et al*.^11^ Access to summary statistics from the FinnGen resource can be obtained at https://www.finngen.fi/en/access_results/].
