# Supplementary material for: Hyperglycaemia is a causal risk factor for upper limb pathologies
Source: Int J Epidemiol. 2024 Jan 10;53(1):dyad187. doi: 10.1093/ije/dyad187 (PMC10859137; doi:10.1093/ije/dyad187)
Supplement: dyad187_Supplementary_Data [file dyad187_supplementary_data.zip › ije-2023-01-0016-File006.docx]

**Figure S1**


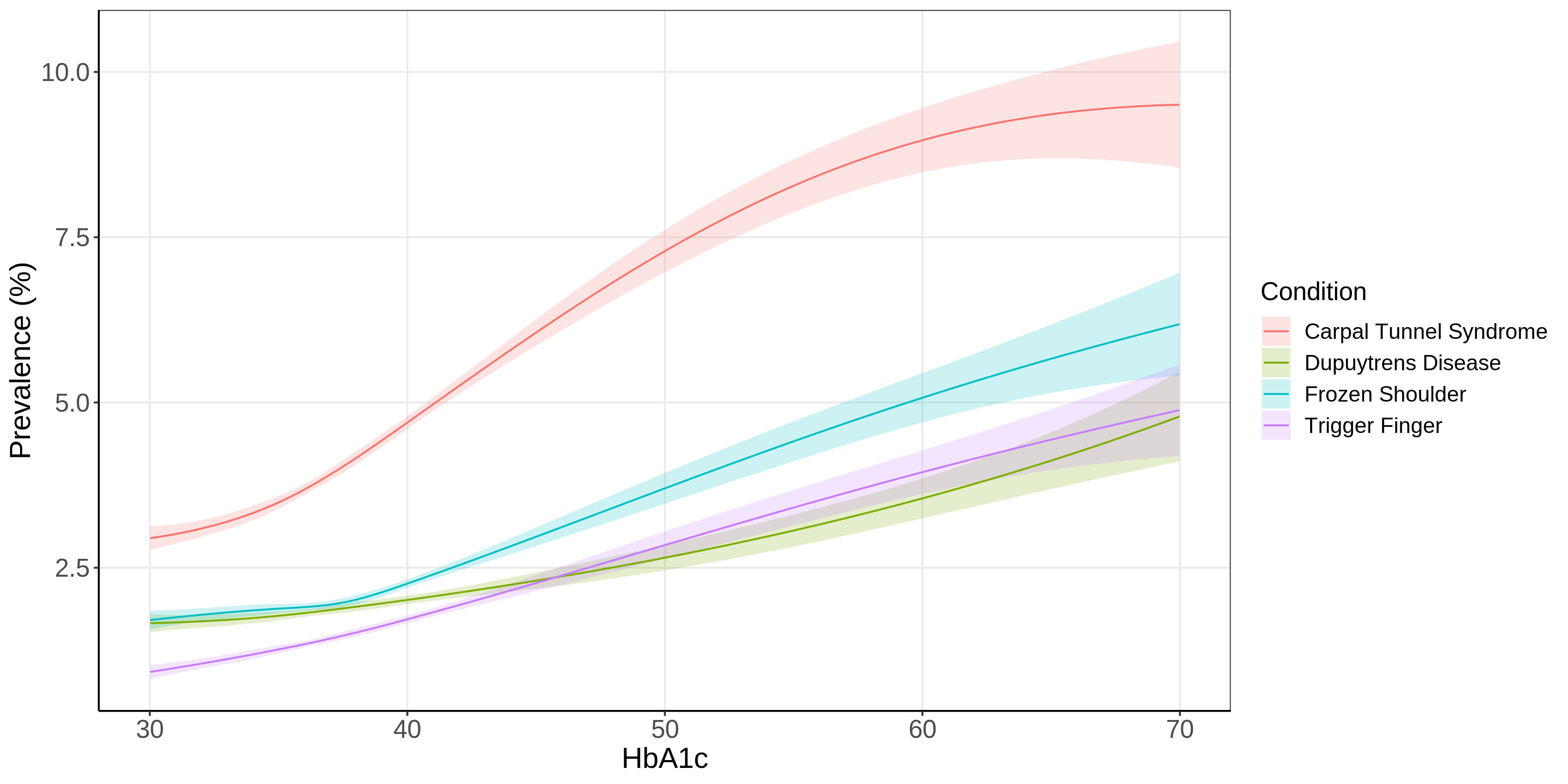


*Plot showing non-linear associations between HbA1c and musculoskeletal conditions, calculated using spline logistic regression.*

**Figure S2**


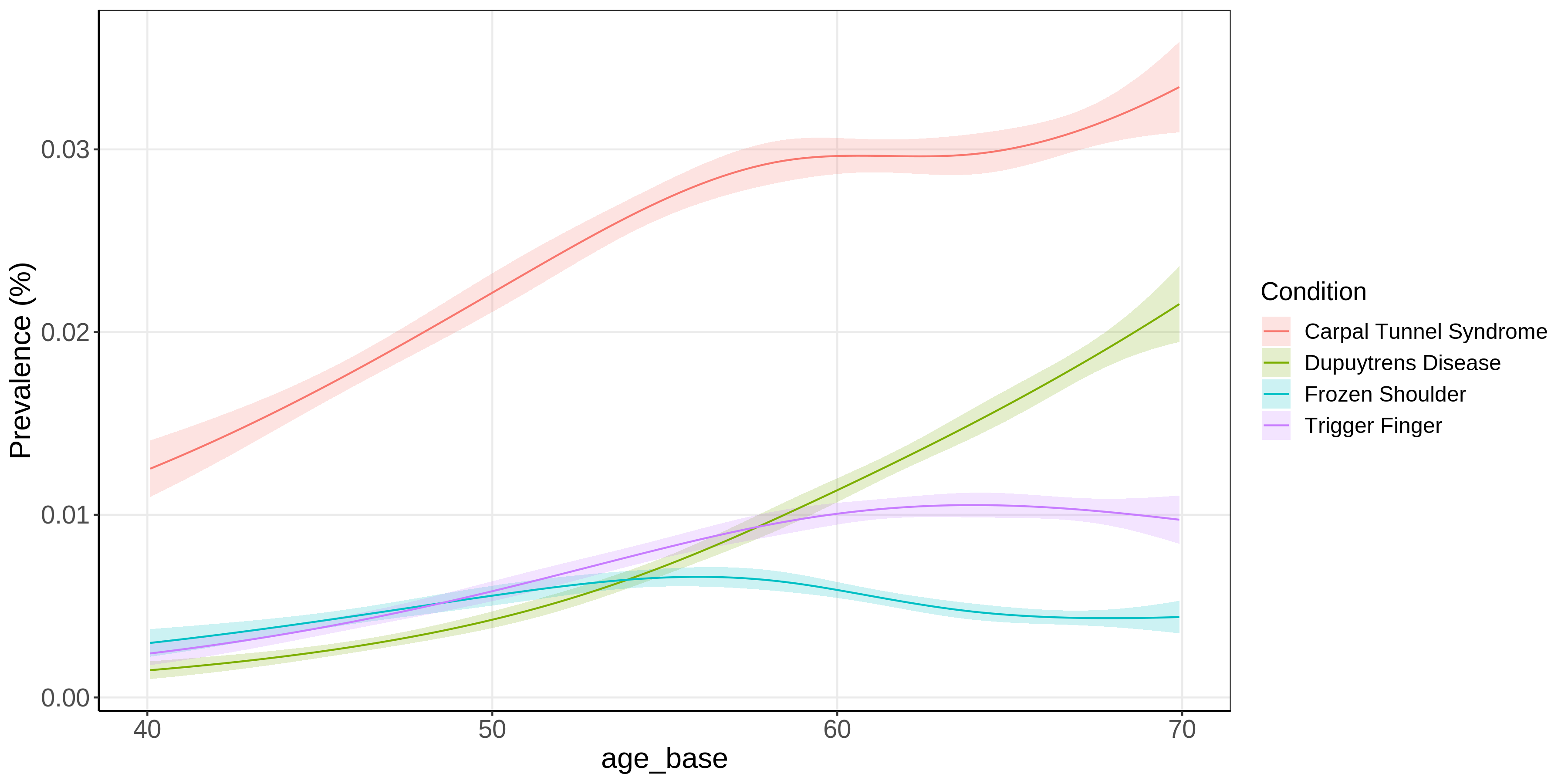


*Plot showing non-linear associations between age and musculoskeletal conditions, calculated using spline logistic regression.*

**Figure S3**


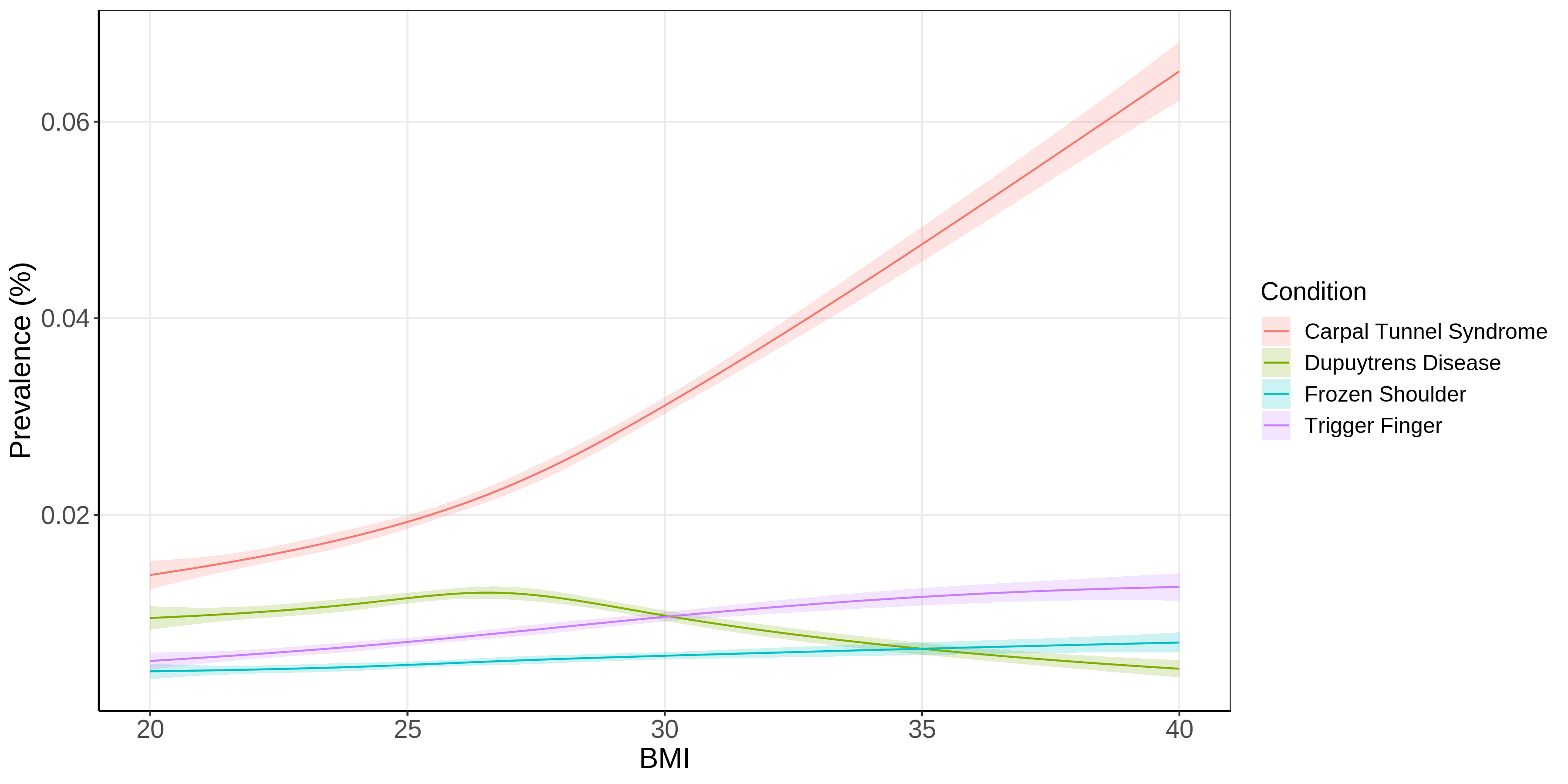


*Plot showing non-linear associations between BMI and musculoskeletal conditions, calculated using spline logistic regression.*
